# Determination of Biogenic Amines in Different Parts of *Lycium barbarum* L. by HPLC with Precolumn Dansylation

**DOI:** 10.3390/molecules26041046

**Published:** 2021-02-17

**Authors:** Yun Ai, Yan Ni Sun, Li Liu, Fang Yuan Yao, Yan Zhang, Feng Yi Guo, Wen Jie Zhao, Jian Li Liu, Ning Zhang

**Affiliations:** 1 Key Laboratory of Resource Biology and Biotechnology in Western China, Ministry of Education, School of Life Sience, Northwest University, Xi’an 710069, China; aiyun-vip@163.com (Y.A.); sunyanni@nwu.edu.cn (Y.N.S.); 18792597747@163.com (L.L.); yaoyao612525@163.com (F.Y.Y.); z18391092679@163.com (Y.Z.); guofengyii@163.com (F.Y.G.); 17835424667@163.com (W.J.Z.); jlliu@nwu.edu.cn (J.L.L.); 2 Xi’an Institute for Food and Drug Control, Xi’an 710054, China

**Keywords:** biogenic amines, *Lycium barbarum* L., HPLC, derivatization

## Abstract

The aim of this work was to characterize biogenic amines (BAs) in different parts of *Lycium barbarum* L. using HPLC with dansyl chloride derivatization, and jointly, to provide referential data for further exploration and utilization of *Lycium barbarum* L. The linear correlation coefficients for all BAs were above 0.9989. The limits of detection and quantification were 0.015–0.075 and 0.05–0.25 μg/mL, respectively. The relative standard deviations for the intra-day and inter-day precision were 0.66–2.69% and 0.91–4.38%. The described method has good repeatability and intermediate precision for the quantitative determination of BAs in different parts of *Lycium barbarum* L. Satisfactory recovery for all amines was obtained (79.3–110.3%). The result showed that there were four kinds of BAs. The highest putrescine content (20.9 ± 3.2 mg/kg) was found in the flower. The highest histamine content (102.7 ± 5.8 mg/kg) was detected in the bark, and the highest spermidine (13.3 ± 1.6 mg/kg) and spermine (23.7 ± 2.0 mg/kg) contents were detected in the young leaves. The high histamine (HIS) content in the bark may be one of the reasons why all of the parts of *Lycium barbarum* L., except the bark, are used for medicine or food in China. Meanwhile, the issue of the high concentration of HIS should be considered when exploiting or utilizing the bark of *Lycium barbarum* L.

## 1. Introduction

Biogenic amines (BAs), such as tyramine (TYR), methylamine (MET), histamine (HIS), putrescine (PUT), cadaverine (CAD), tryptamine (TRY), phenylethylamine (PEA), spermine (SPM), and spermidine (SPD), are endogenous and indispensable components to living cells, playing important roles in cell proliferation and differentiation, regulation of nucleic acid function, protein synthesis, brain development, and nerve growth and regeneration [1]. Moreover, they have many biological activities, such as vasoconstriction [2], vasodilation [3], antioxidant [4], and promoting longevity [5]. For polyamines PUT, SPD, and SPM, their intracellular concentrations decline during human aging, and administration of SPD markedly extended the lifespans of yeast, flies, worms, and human immune cells. The SPD administration potently inhibited oxidative stress in aging mice. In aging yeast, spermidine treatment triggered epigenetic deacetylation of histone H_3_ through the inhibition of histone acetyltransferases (HAT), suppressing oxidative stress and necrosis. Conversely, depletion of endogenous polyamines led to hyperacetylation, generation of reactive oxygen species, early necrotic death, and decreased lifespan [5]. In recent years, it was found that SPD and SPM potently inhibit oxidative stress in aging mice, which can increase the activity of antioxidant enzymes, reduce the accumulation of free radicals, improve the skeletal muscle cell membrane metabolism and anti-injury ability, and significantly delay the occurrence of mouse fatigue [6,7]. BAs in low concentrations are essential for many physiological functions; however, they can cause a variety of side effects in high concentrations, including rashes, headaches, nausea, hypo- or hyper-tension, cardiac palpitations, intracerebral hemorrhages, and anaphylactic shock, especially when alcohol or monoamine oxidase inhibitors are ingested at the same time [8]. Among the BAs, the toxicity of each amine is different, but HIS and TRY are the most toxic. HIS poisoning has a short incubation period, ranging from minutes to hours after ingestion. Symptoms include headache, facial flushing and sweating, rash and itching, nausea, vomiting, diarrhea, and heart palpitations [9]. TYR overdose may result in a hypertensive crisis, usually accompanied by a severe headache and sometimes intracranial hemorrhage and other neurological sequelae, cardiac failure, and pulmonary edema [10]. It was reported that 3 mg of PEA can cause migraine headaches in susceptible individuals [1]. PUT and CAD have not demonstrated direct adverse health effects, but they are known to enhance HIS toxicity by inhabiting HIS metabolizing enzymes [1]. The permissible limit of 50 mg/kg of HIS has been proposed by the US Food and Drug Administration (US FDA)—differently, 100 mg/kg was proposed by the European Union, South Africa, Canada, and Switzerland, and 200 mg/kg by Australia [11,12,13]. The acceptable level of TYR is 100–800 mg/kg and for PEA it is 30 mg/kg [1,4]. No recommendations have been suggested for PUT and CAD. Although estimating the total toxic dose of individual BAs is very difficult, Shalaby and Valsamaki et al. have stated that the safe sum of HIS, TRY, PUT, and CAD should not significantly exceed 900 mg/kg [14,15]. Therefore, the study of BAs is of great interest due to their activities and toxicological threats.

*Lycium barbarum* L. (*L. barbarum*) is a perennial bush produced mostly in Ningxia Hui Autonomous Region of China. The Compendium of Materia Medica in the Ming Dynasty recorded: the leaves of *L. barbarum* are called Tianjingcao; the flowers are called Changshengcao; the fruits are called Gouqizi (Lycii Fructus); the root bark is called Digupi (Cortex Lycii Radicis) [16]. In Traditional Chinese Medicine (TCM), the roots, stems, leaves, flowers, and fruits of *L. barbarum* are used as medicine [17]. The fruit of *L. barbarum*, known as the wolfberry, is a well-known product used for medicinal and food purposes in China [18,19]. It was first recorded in the book of “Shennong’s Classic of Materia Medica” and described as making the body stronger, resistant to aging, and resistant to cold and heat, and enhancing physical fitness. It was listed in the Chinese Pharmacopoeia (2020). The dried fruit of *L. barbarum*, widely used as a very important commercial crop, has been developed and used in different kinds of products, including medlar wine, tea, drinks, health products, and cosmetics [20]. It is also used in food such as stewed soup and porridge. The fruit of *L. barbarum* also has been used for preventing and treating various diseases in East Asia for over 2000 years with excellent efficacy in nourishing the liver and kidney, brightening the eyes [21], and providing anti-aging and cytoprotective effects [22]. The leaves of *L. barbarum*, which are recorded in the book of Chinese Materia Medica, have effects of tonifying the kidney and providing a beneficial essence, removing heat and quenching thirst, nourishing the liver and improving eyesight, and promoting longevity [16]. Modern pharmacology shows that it has antioxidant, anti-aging, hypoglycemic, hypolipidemic, anti-tumor, antibacterial, anti-hypoxic, and anti-fatigue activities [23,24,25]. The leaves of *L. barbarum* also have been used as a daily health food, a health-enhancing herbal tea, and vegetables [26]. The leaves of *L. barbarum* and cactus are reportedly used to make a hypoglycemic health drink to control blood glucemin [27]. Stems and leaves of *L. barbarum* and garlic as mixed raw materials can be made as a composite function beverage [28]. The flower of *L. barbarum* has antioxidant, neuroprotective, and antibacterial effects [23]. The root of *L. barbarum* has anti-free radical activity, has immune regulation activity, lowers blood pressure, regulates blood lipids, is hypoglycemic, and is antibacterial [25]. Therefore, it is valuable to study the different parts of *L. barbarum* for its better exploitation and utilization.

For the study of the composition of *L. barbarum*, most studies have reported flavonoids, alkaloids, terpenoids, polysaccharides, peptides, phenolic amides, traceelements, and amino acids in the fruit or leaves of *L. barbarum* [20,29,30]. Ma Baolong et al. [31] successfully established a method for detecting chloride derivatives of SPM and SPD in *L. barbarum* leaves with high performance liquid chromatography (HPLC), but they did not provide examples, nor did they give specific contents. To the best of the authors’ knowledge, there are no reports available about specific BA contents in the different parts of *L. barbarum*.

A variety of methods have been applied for the determination of BAs in foods, such as electrochemical biosensor methods [32], thin layer chromatography (TLC) [33], gas chromatography (GC) [34], ion chromatography (IC) [35], and reverse-phase HPLC (RP-HPLC) [36]. Since most BAs lack satisfactory absorption and significant fluorescence characteristics, chemical derivatization is usually performed to increase the sensitivity in determining BAs. O-phthalaldehyde (OPA) [37] and dansyl chloride (Dns-Cl) [38] are mostly used. The major disadvantage of OPA is that it only reacts with primary amines. However, Dns-Cl forms derivatives not only with primary but also with secondary amines. Their products are more stable than those formed by using OPA [39]. BAs were characterized in Chinese Tonic-Qi herbs, male silkworm moths, and rat plasma using HPLC with Dns-Cl derivatization in our previous studies [40,41,42]. This method has good repeatability and precision for the quantitative determination of BAs. This work aimed to characterize BAs in the different parts of *L. barbarum* using HPLC with Dns-Cl derivatization and to provide referential data for further exploration and utilization of *L. barbarum*. The identification of dansylated BAs was confirmed by HPLC-UV and ESI-Q-TOF-MS.

## 2. Results and Discussion

### 2.1. Preparation of Samples

In order to optimize the solution extracted, trichloroacetic acid, perchloric acid, and hydrochloric acid were employed to compare the extraction efficiency of BAs from *L. barbarum*. The best results came from the use of hydrochloric acid as the extraction reagent with an optimal concentration of 1 M. To determine low concentrations of BAs in the complex samples, it can be of great importance to remove interfering constituents in order to obtain the baseline separated peaks. Therefore, the amine was concentrated by liquid–liquid extraction and the interference components were removed. A total 10 mL of 1 M HCl extraction was collected, and the pH was adjusted to 12 to obtain free amines. The n-butyl alcohol-dichloromethane (1:1, *v/v*) was used to extract the free amines. One milliliter of 1 M HCl was added to the organic fractions before evaporation in order to obtain the stable amine hydrochloride.

BAs can be formed stable amine hydrochloride when extracted with 1 M HCl. An additional liquid–liquid extraction was employed to remove highly polar or ionic compounds. After organic solvent evaporation, the dry residue was dissolved in 0.1 M HCl (1 mL) for enrichment finally. The chemical constituents of *L. barbarum* are very complex, including anthocyanins, flavonoids, polysaccharides, fatty acids, amino acids, sugars, and some trace elements. Extraction of BAs from samples and pre-processing are important steps prior to the derivatization. Most of the methods available in the literature for BA determination involve an acidic extraction from a solid matrix; organic solvents are seldom used. The choice of acid has to be related to the characteristics of the matrix to be analyzed. Many compounds that might interfere can be eliminated in this step. In our previous study, an optimization of the extraction procedure was carried out by investigating the influences of extraction solvent (acid choice), extraction method, and extraction time on the outputs of the extraction [40,41,42]. To determine low concentrations of BAs in crude extracts from plant tissues, it can be of great importance to remove interfering compounds; purification is necessary. Therefore, an additional liquid–liquid extraction was employed to remove highly polar or ionic compounds from the plant extract. BAs were dissociated after adding NaOH, and extracted by an organic solvent which can remove water-soluble impurities (amino acids and sugar). After organic solvent evaporation, the dry residue was dissolved in 0.1M HCl (1 mL) for enrichment. This method has good repeatability, precision, and recovery for the quantitative determination of BAs.

### 2.2. Derivatization Procedure

To optimize the derivatization procedure, different amounts of Dns-Cl (between 3 and 10 mg/mL) and reaction times (from 30 to 60 min) were used to derivatize the 11 amines at different temperatures (between 25 and 80 °C). It was found that a 45 min derivatization at 60 °C by using 5 mg/mL Dns-Cl was sufficient to produce a quantitative conversion of the amines into stable Dns-Cl derivatives. As the dansylation reaction requires an alkaline state, the pH of the buffer is the main influence for dansylation on these 11 determined amines. Upon comparison of different pHs of the NaHCO_3_/Na_2_CO_3_ buffer from 8.16 to 11.0, a suitable pH of 10.0 was determined. An addition of ammonia is necessary to quench an excess of Dns-Cl, and a reaction for 30 min is sufficient to stop the dansylation. Experiments have shown that the dansyl derivative is stable for more than 24 h at room temperature.

### 2.3. Identification of the BAs in L. barbarum

**Figure 1:**
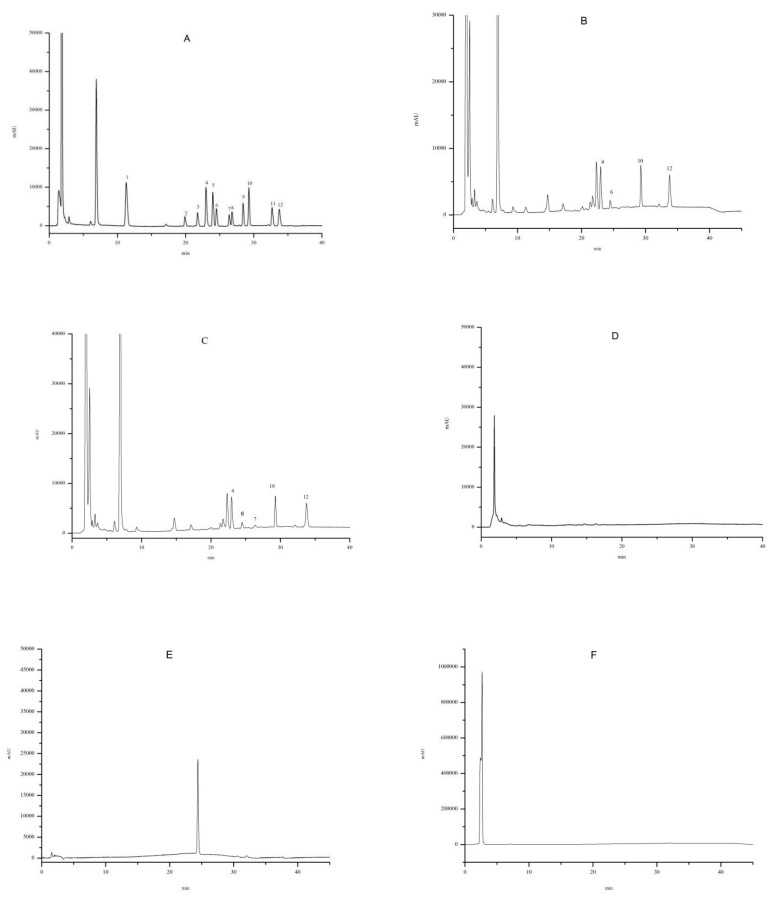
**(A**): Standard solution of BAs (10 µg/mL each); (**B**): *L. barbarum* leaves sample (IS unspiked); (**C**): *L. barbarum* leaves sample; (**D**): *L. barbarum* leaves sample (Dns-Cl unspiked); (**E**): Dns-Cl solution (20 µg/mL); (**F**): control group: 10 mL 1 M HCl replacing the 10 mL sample extract derived with Dns-Cl. Chromatographic peaks: (1) MET; (2) TRP; (3) PEA; (4) PUT; (5) CAD; (6) HIS; (7) DMP; (8) 5-HT; (9) TYR; (10) SPD; (11) DA; (12) SPM.

In the analysis of *L. barbarum* samples, the BAs peak identification was based on the comparison between the retention times of standard compounds and was confirmed by ESI-Q-TOF-MS. The chromatogram of a standard solution of dansylamines shown in Figure 1A was obtained using the gradient profile described in the Section 3.5. There was no overlap between each standard amine. The high content of acetonitrile (ACN) in the mobile phase within the twenty-fifth and thirty-fifth minutes is a necessity due to the slow movement of SPD and SPM in the column. After SPM elution, the composition of the mobile phase returned within 5 min to the initial composition. All of the standard amine peaks appeared within 35 min. The detection peaks of different parts of *L. barbarum* samples could be well separated with no endogenous interference observed at the retention times of both the analyte and the internal standard (IS). The sample of young leaves, as a representative one, is shown in Figure 1B–D. Major side-products of the dansyl reaction were eluted for 2–10 min and good separation was achieved from derivatized amines. The control group was detected using 10 mL of 1 M HCl to replace the 10 mL of sample extract derived with Dns-Cl and shown in Figure 1F.

**Figure 2:**
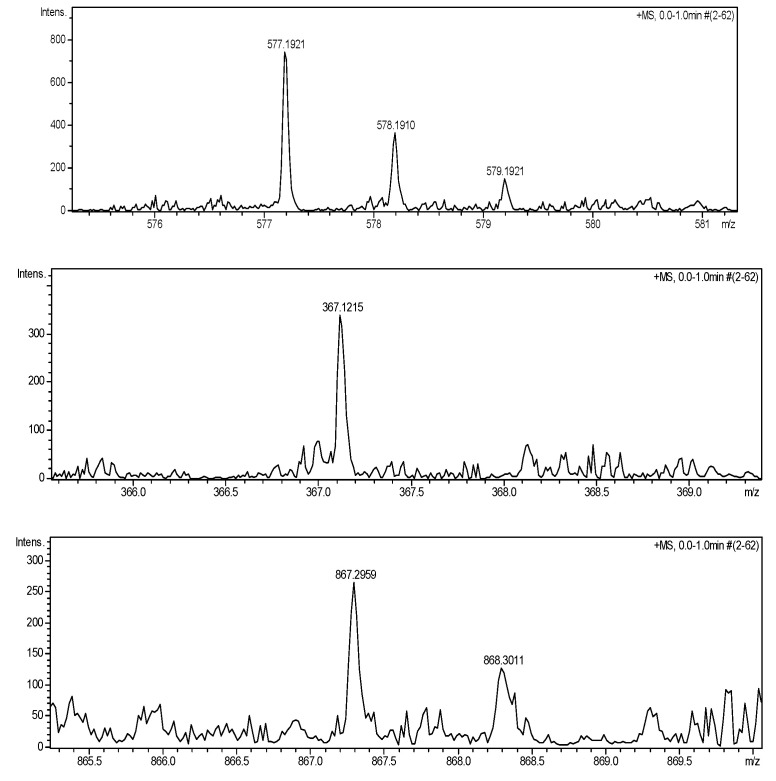
MS spectra of dansylated BAs.

Under the MS conditions, dansylated BAs produced stable and intense [M + Na]^+^ ions seen in Figure 2 and Table 1. However, the product ion of SPM was not found, though the dansylated SPM was analyzed alone or the concentration of the analyte was increased. The dansylated SPM (MW: 1157) may be too large to detect under the MS conditions. SPM was identified by changing the mobile phase and the spiked standards [40].

### 2.4. The Matrix Effect

The matrix effect was assessed by the slope comparison method as performed by Flores and Jia groups [43,44]. The slope ratio of the matrix curve to the neat solution curve was calculated; the ratio value of 1.0 indicated no matrix effect. The results showed that most of the ratio values were close to 1.0, implying that there were no significant matrix effects in relatively complex plant matrices. Since no significant matrix effects were found for the relatively complex plant matrices, the proposed method might be applicable for the detection of BAs in other complex samples in the future.

### 2.5. Method Validation

The results are summarized in Table 2. Good fitting with the linear model for the response (R^2^: 0.9989–0.9999) of each analyte was observed in the concentration ranges of 0.10–10, 0.25–10, and 0.10–50μg/mL. The LOD and LOQ were in the ranges of 0.015–0.075 and 0.05–0.25 μg/mL, respectively. The relative standard deviations (R.S.Ds.) for the intra-day and inter-day precision were 0.66–2.69% and 0.91–4.38%, respectively. These results confirmed the good repeatability and intermediate precision of the described method. Satisfactory recovery for all amines was obtained (79.3–110.3%); young leaves sample, as a representative, is shown in Table 2.

### 2.6. Distribution of the BAs in L. barbarum

Table 3 lists the BAs in different parts of *L. barbarum*.

As shown in Table 3, MET, TRP, PEA, CAD, 5-HT, TRY, and DA were not found in detectable concentrations in any of the samples. PUT, HIS, SPD, and SPM were found in all parts of *L. barbarum*, except that HIS was not found in detectable concentrations in the fruit.

The content of BAs was determined and compared in different parts of *L. barbarum*. The PUT content was the highest in the flower (20.9 ± 3.2 mg/kg), followed by young leaves, young stem, mature stem, root, bark, mature leaves, and fruit. The PUT content of the flower and young leaves, young stem, mature stem, root, and bark did not significantly differences among the six groups (*p* >0.05); however, there were significant differences in PUT content among flower, mature leaves, and fruit (*p* < 0.05).

The highest HIS content was detected in the bark (102.7 ± 5.8 mg/kg), followed by mature stem (23.5 ± 2.2 mg/kg), root (17.0 ± 3.2 mg/kg), young stem (10.2 ± 1.8 mg/kg), young leaves (5.6 ± 0.9 mg/kg), mature leaves (2.6 ± 0.3 mg/kg), and flower (2.3 ± 0.4 mg/kg). HIS was not found in detectable concentrations in the fruit. The content of HIS in the bark was much higher than that in the other seven groups, and the difference was significant (*p* < 0.05). The HIS content in the mature stem was the second, with no significant difference from root, but there was a significant difference between the mature stem and young leaves, mature leaves, young stem, and flower (*p* < 0.05). The contents of HIS in mature leaves, young leaves, young stem, flower, and root were very low and were not significantly different (*p* > 0.05). The HIS content in the bark (102.7 ± 5.8 mg/kg) was higher than the limit standard (50 mg/kg by the US FDA, 100 mg/kg by the European Union, South Africa, Canada, and Switzerland). Considering the toxicity of HIS [1,9], it is necessary to pay attention to the high toxicity of HIS in the bark. The experimental result suggests that high HIS should be considered when exploiting or utilizing the bark of *L. Barbarum*. The HIS contents in young leaves, mature leaves, young stem, flower, and root were not significantly different, and were lower than regulated limits, so they can be used safely. The Compendium of Materia Medica recorded that the root, stem, leaves, flower, and fruit are used as medicine; only the bark is not. The bark is also not used for food in China. The high content of HIS in the bark may be one of the reasons.

Upon comparison with the SPD contents in different parts of *L. barbarum*, the highest amount of SPD was detected in the young leaves (13.4 ± 1.6 mg/kg), followed by mature leaves (10.7 ± 1.5 mg/kg) and flower (10.7 ± 2.1 mg/kg), all of which had significantly higher contents than the other parts. The difference between these three groups is not obvious (*p* > 0.05), but there was significant difference from other groups (*p* < 0.05). The highest SPM content was found in the young leaves, followed by young stem, mature leaves, fruit, flower, bark, mature stem, and root. Compared with the contents of SPM in different parts of *L. barbarum*, the contents of SPM in young leaves (23.7 ± 2.0 mg/kg), young stem (13.3 ± 1.4 mg/kg), mature leaves (9.4 ± 1.1 mg/kg), and fruit (6.3 ± 1.2 mg/kg) were significantly higher than those in the other parts, and the content of SPM in young leaves was the highest. A significant difference existed between the content of SPM in young leaves and the contents in the other seven groups (*p* < 0.05). The SPM contents in mature leaves and young stem were higher than in mature stem, bark, flower, fruit, and root, significantly (*p* < 0.05). No significant difference for SPM content was observed between mature leaves and young stem (*p* > 0.05).

## 3. Materials and Methods

### 3.1. Chemicals and Reagents

BAs, 2-phenylethylamine (PEA), methylamine (MET), putrescine dihydrochloride (PUT), cadaverine dihydrochloride (CAD), tryptamine (TRY), histamine dihydrochloride (HIS), serotonin hydrochloride (5-HT), tyramine hydrochloride (TYR), dopamine hydrochloride (DA), spermidine trihydrochloride (SPD), and spermine tetrahydrochloride (SPM) were obtained from Sigma-Aldrich (St. Louis, MO, USA). IS, 1,7-diaminoheptane (DMP) was supplied by Sigma-Aldrich (Steinheim, Germany). Dansyl chloride (Dns-Cl) from Sigma-Aldrich (USA) was used as a derivatization reagent. The HPLC grade acetonitrile (ACN) was purchased from Tedia (Fairfield, CT, USA). Deionized water was produced by a MILLPAK Reagent Water System (Millipore, MA, USA). All other reagents were of analytical grade.

### 3.2. Preparation of Standard Solutions

The mixed stock solution, containing 0.1 mg/mL of each individual amine, was prepared in 0.1 M HCl and stored at 4 °C. Standard solutions were prepared by diluting the stock solution and used to obtain calibration curves. A separate IS stock solution, containing 0.1 mg/mL DMP, was made in an analogous way. Dns-Cl standard solution was prepared in acetone and stored at 4 °C.

### 3.3. Preparation of Sample Solution

*L. barbarum* samples were collected from Xi’an Town, Zhongwei City, NingXia Hui Autonomous Region of China (31 August), and were categorized as young leaves, mature leaves, young stems, mature stems, main stem bark, flowers, fruit, and roots. Each sample was dried in the shade according to the traditional drying method of *L. barbarum* fruit (known as the wolfberry) [45].

The different parts were collected from multiple trees, then cut into small pieces (0.5 cm * 0.5 cm) and mixed, and then 3 samples (5 g each sample) were weighed randomly. Each sample was transferred into a 50 mL centrifuge tube, followed by the addition of 20 mL of 1 M HCl and 0.1 mL of IS working solution (0.1 mg/mL). The mixture was extracted in a constant-temperature shaker (SHA-C, China) for 60 min and then centrifuged (3600 rpm) for 10 min. The supernatant was collected with the residue extracted twice with the same volume of 1 M HCl. All supernatants were combined and filtered through a filter paper into a 50 mL brown volumetric flask. The final volume was adjusted to 50 mL with 1 M HCl. A 10 mL aliquot of sample extract was transferred to a 50 mL centrifuge tube and adjusted to pH 12 with 2 M NaOH. The mixture was extracted with 10 mL n-butyl alcohol-dichloromethane (1:1, *v/v*), vortex-mixed for 5 min and then centrifuged for 10 min at 3600 rpm. After separating the two phases, the lower organic phase was transferred to an evaporating dish. The extraction was repeated three times. The 1 mL of 1 M HCl was added to the combined organic fractions and evaporated to dryness under nitrogen in a water bath at 40 °C. The dried residue was dissolved in 1 mL of 0.1 M HCl, and 0.5 mL solution was collected for derivatization. The residue was used for the blank group. The control group was carried out with a 10 mL 1 M HCl replaced 10 mL extract, then followed the rest steps.

### 3.4. Derivatization Procedure

The 0.5 mL of dilute standard solution of the amines was mixed with 20 μL DMP (0.1 mg/mL), 0.5 mL of Dns-Cl (5 mg/mL in acetone), and 1 mL of Na_2_CO_3_-NaHCO_3_ pH 10 buffer. The mixture was heated in a water bath at 60 °C for 45 min. The excess Dns-Cl was quenched by adding 100 μL of ammonia in the dark at room temperature. After 30 min, the mixture was adjusted to 5 mL with ACN. The solution was filtered through a 0.45 μm membrane filter and injected onto the chromatographic column for determination. For each of the extracted samples, a dansylation reaction was performed as described earlier for the standard solution, except that IS was not added.

### 3.5. Instrumentation and Conditions

The HPLC determination of dansyl derivatives of BAs was performed with a Shimadzu liquid chromatography system coupled with a system controller SCL-10A-Vp, two pumps (LC-10A-Vp), a DAD detector SPD-M-10A-Vp, a degasser GT-154, and a Rheodyne injector with a 20 μL loop (Tokyo, JPN). Separation was achieved using a Shimadzu shim-pack C18 analytical column (250 mm × 4.6 mm, Intenal Diameter, 5 μm) and protected with a Shimadzu extend C18 guard column (12.5 mm × 4.6 mm, Intenal Diameter, 5 μm) maintained at 30 °C. The mobile phase solution A was ultra-pure water and the mobile phase solution B was ACN. Gradient elution was from 45–50 % A for 0–7 min; 50–10% A for 8–25 min; 10% A for 26–35 min and 10–45 % A for 36–40 min at the flow rate of 0.8 mL/min. The detector was set at 254 nm; 20 μL of dansyl derivative was injected onto HPLC for analysis. 

Mass spectrometry was carried out on a micrOTOF-Q II mass spectrometer instrument (Bruker Daltonics, Bremen, Germany). The conditions of MS analysis in the positive ion mode were as follows: dry gas; flow rate, 4.0 mL min^−1^; dry heater temperature, 180 ℃; scan range, 50–3000 *m/z*; capillary voltage, 4500 V; nebulizer press, 0.4 Bar.

### 3.6. Method Validation

The analytical method was validated in terms of its linearity, limit of detection (LOD), limit of quantification (LOQ), precision, and recovery. The calibration curves were generated by plotting the peak-area ratios of the analyte to the IS relative to the analyte concentrations against the seven concentrations of the standard mixtures. The LOD and LOQ were calculated based on the signal-to-noise ratios (S/N) of 3 and 10, respectively. Five replicates of the same sample were analyzed within one working day to determine the intra-day precision. The sample was continuously measured for three days, repeatedly, five times a day, to determine the inter-day precision. Recovery studies were carried out by adding 4 mL of a mixed standard solution at three different concentrations (1, 5, and 10 μg/mL) to three young leaves samples, the amine content of which had been predetermined. The standard solution was added to the sample in the first step (extracted with 1M HCl) [40].

### 3.7. Evaluation of the Matrix Effect

As it is very difficult to find blank plant matrix samples free of BAs, the slope comparison method was used instead to evaluate the matrix effect for this study. The leaf sample extracts, which were spiked with appropriate amounts of mixed standard solutions, as done for the apparent recovery measurement described in Section 3.6, were used to construct standard addition calibration curves. Then, the slopes of the calibration curves from the standard addition experiments were compared with the slopes obtained from the pure aqueous standards at the same concentration levels.

### 3.8. Statistical Analysis

The calculation of linearity relationship between peak-area ratios of analyte to the IS and concentrations of analyte were adopted by weighted-least-squares linear regression. SPSS 20.0 was used for all statistical tests. Data were presented as the mean ± standard deviation (SD). Single factor analysis of variance (One-way ANOVA) and Tukey’s post hoc test were used for comparisons between groups. One-way ANOVA was used to determine group differences. The Tukey post hoc test was used for multiple comparisons among groups. A *p*-value < 0.05 was considered statistically significant.

## 4. Conclusions

In this work, a method involving HPLC with precolumn derivatization for determination of 11 BAs in *L. barbarum* was developed. This is the first report showing that different parts of *L. barbarum* contain PUT, HIS, SPD, and SPM. The PUT content was the highest in the flowers; the HIS content was the highest in the bark; the SPD and SPM contents were the highest in the young leaves. HIS was not found in the fruit and its content in the leaves was very low, which makes them safe when used in drugs and food. The high HIS content in the bark may be one of the reasons why all of the parts of *L. barbarum,* except the bark, are used for medicine or food in China. Meanwhile, the issue of high concentration of HIS should be considered when exploiting or utilizing the bark of *L. Barbarum*.

## Figures and Tables

**Table 1:** Characteristic mass fragments of dansylated BAs.

BAs	Dns-BAs	[M + 23]^+^ (*m/z*)	[M + 23]^+^ (*m/z*) found
PUT	Dns_2_-PUT	577.1914	577.1921
HIS	Dns-HIS	367.1199	367.1215
SPD	Dns_3_-SPD	867.3003	867.2959

**Table 2:** Sample characteristics obtained with HPLC analysis.

Biogenic Amines	Linear Range (μg/mL)	R^2^	LOD (μg/mL)	LOQ (μg/mL)	Intra-Day Precision (%RSD) ( *n* = 5)	Inter-Day Precision (%RSD) ( *n* = 5)	Recovery (Young Leaves Samples) (% mean ± SD, *n* = 3)		
							1 μg/mL	5 μg/mL	10 μg/mL
MET	0.10–10	0.9999	0.015	0.05	0.98	2.54	89.1 ± 4.9	91.7 ± 1.3	86.5 ± 1.1
TRP	0.10–10	0.9990	0.030	0.10	0.81	1.53	79.3 ± 4.6	92.1 ± 1.2	91.7 ± 1.0
PEA	0.25–10	0.9997	0.075	0.25	1.01	1.73	92.6 ± 5.3	94.3 ± 1.6	90.5 ± 1.2
PUT	0.10–50	0.9996	0.015	0.05	2.69	4.38	88.2 ± 5.4	90.6 ± 1.6	88.6 ± 1.2
CAD	0.10–10	0.9998	0.030	0.10	0.93	1.67	86.3 ± 4.3	93.6 ± 1.6	95.5 ± 1.1
HIS	0.10–10	0.9993	0.030	0.10	1.27	2.39	89.5 ± 4.7	91.2 ± 1.2	87.9 ± 1.1
5-HT	0.25–10	0.9989	0.075	0.25	1.33	2.06	110.3 ± 5.1	88.1 ± 2.0	87.7 ± 1.7
TYR	0.25–10	0.9997	0.075	0.25	0.66	0.91	86.5 ± 4.1	88.2 ± 1.1	89.4 ± 1.3
SPD	0.10–10	0.9997	0.030	0.10	1.42	2.82	89.7 ± 5.7	92.6 ± 1.9	90.4 ± 1.4
DA	0.25–10	0.9989	0.075	0.25	1.09	2.43	93.1 ± 4.8	94.1 ± 2.5	88.5 ± 1.7
SPM	0.10–10	0.9996	0.030	0.10	2.07	3.31	96.5 ± 4.4	94.2 ± 1.4	90.1 ± 1.1

R^2^: square of regression coefficient; LOD: limit of detection; LOQ: limit of quantification; RSD: relative standard deviation.

**Table 3:** Content of BAs in different parts of *L. barbarum*. (*n* = 3).

Samples	Concentrations of BAs (mg/kg) (Mean ± SD)										
	MET	TRP	PEA	PUT	CAD	HIS	5-HT	TYR	SPD	DA	SPM
Young leaves	ND	ND	ND	12.94 ± 1.8 ^ab^	ND	5.6 ± 0.9 ^c^	ND	ND	13.3 ± 1.6 ^a^	ND	23.7 ± 2.0 ^a^
Mature leaves	ND	ND	ND	2.3 ± 0.4 ^d^	ND	2.6 ± 0.3 ^c^	ND	ND	10.7 ± 1.5 ^abc^	ND	9.4 ± 1.1 ^bc^
Young stem	ND	ND	ND	10.4 ± 1.4 ^abc^	ND	10.2 ± 1.8 ^c^	ND	ND	4.3 ± 0.8 ^bc^	ND	13.3 ± 1.4 ^b^
Mature stem	ND	ND	ND	5.2 ± 1.1 ^acd^	ND	23.5 ± 2.2 ^b^	ND	ND	3.6 ± 0.6 ^bc^	ND	2.4 ± 0.4 ^d^
Bark	ND	ND	ND	3.0 ± 0.5 ^ad^	ND	102.7 ± 5.8 ^a^	ND	ND	3.0 ± 0.5 ^c^	ND	2.7 ± 0.4 ^d^
Flower	ND	ND	ND	20.9 ± 3.2 ^a^	ND	2.3 ± 0.4 ^c^	ND	ND	10.7 ± 2.1 ^abc^	ND	4.2 ± 0.7 ^d^
Fruit	ND	ND	ND	1.3 ± 0.3 ^d^	ND	ND	ND	ND	2.9 ± 0.1 ^bc^	ND	6.3 ± 1.2 ^cd^
Root	ND	ND	ND	5.2 ± 0.9 ^abcd^	ND	17.0 ± 3.2 ^bc^	ND	ND	3.0 ± 0.3 ^bc^	ND	0.9 ± 0.1 ^d^

ND: not detected. Different letters (such as a and b) indicate differences between period groups (*p* < 0.05), identical letters (including a and ab) indicate no differences between period groups, and no letters indicate no differences among all eight period groups.

## Data Availability

HPLC dates are available from the authors.

## Sample Availability

Samples of *L. barbarum* are available from the authors.

## Abbreviation

BAs: biogenic amines; MET, methylamine; PEA, 2-phenylethylamine; TYR, tyramine; HIS, histamine; PUT, putrescine; CAD, cadaverine dihydrochloride; TRY, tryptamine; PEA, phenylethylamine; 5-HT, serotonin; DA, dopamine; SPM, spermine; SPD, spermidine; IS, Internal standard; DMP, 1,7-diaminoheptane; ACN, acetonitrile; TLC, thin layer chromatography; GC, gas chromatography; IC, ion chromatography; OPA, O-phthalaldehyde; Dns-Cl, dansyl chloride; LOD, limit of detection; LOQ, limit of quantification; S/N, signal-to-noise ratio; TCM, Traditional Chinese Medicine.
